# Association Between Dietary Protein Intake and Sleep Quality in Middle-Aged and Older Adults in Singapore

**DOI:** 10.3389/fnut.2022.832341

**Published:** 2022-03-09

**Authors:** Clarinda Nataria Sutanto, Wen Wei Loh, Darel Wee Kiat Toh, Delia Pei Shan Lee, Jung Eun Kim

**Affiliations:** Department of Food Science and Technology, Faculty of Science, National University of Singapore, Singapore, Singapore

**Keywords:** sleep quality, sleep duration, tryptophan, Trp:LNAA, plant protein, animal protein, plasma amino acid

## Abstract

Poor sleep has been associated with the increased risk of developing detrimental health conditions. Diet and certain nutrients, such as dietary protein (PRO) may improve sleep. This cross-sectional study aimed to investigate the relationship between PRO intake, their amino acid components, and sources with sleep quality in middle-aged and older adults residing in Singapore. A dataset of 104 healthy subjects between the age of 50 and 75 years old were used. Collected data included 3-day food record and sleep quality [sleep duration, global sleep score (GSS), sleep latency (SL), and sleep efficiency (SE)]. The collected 3-day food records were extracted for PRO, tryptophan (Trp), and large neutral amino acid (LNAA) intake. PRO intake was further categorized into plant and animal PRO. A multivariate multiple linear regression (MLR) was performed to assess the association between PRO intake and sleep quality. Dietary Trp:LNAA ratio was positively associated with sleep duration (β_total_: 108.234 h; *p*: 0.005) after multiple covariates adjustment. Similarly, plant Trp (β_plant_: 2.653 h/g; *p*: 0.020) and plant Trp:LNAA (β_plant_: 54.006 h; *p*: 0.008) was positively associated with sleep duration. No significant associations were observed for both SL and SE. Sleep duration in middle-aged and older Singaporean adults was positively associated with dietary Trp and Trp:LNAA, especially when obtained from plant sources.

## Introduction

Sleep is an integral part of our overall health and wellbeing. Good sleep is often characterized as having a sleep duration of 7–9 h/day, a short sleep latency (SL), and a good sleep efficiency (SE) (1). Sleep deprivation has been reported to negatively impact the metabolic, endocrine, and immune system (2). This may be explained by the higher concentration of cortisol associated with sleep deficiency, which may lead to health issues, such as insulin resistance, immune pressure, and inflammation (3). Consequently, both sleep duration and other sleep quality components have been associated with mental distress and the higher risk of developing chronic disease conditions, such as diabetes, hypertension, and cardiovascular diseases (3). Therefore, it is essential to identify effective strategies to improve sleep-related outcomes and this is especially important in the aging population, where poor sleep quality is more prevalent (1, 4).

Conventional methods to aid sleep typically involve pharmacotherapy or psychological interventions, such as behavioral modification and sleep hygiene techniques. However, the use of drugs can lead to unwanted side-effects and psychological interventions that may be challenging to implement effectively (5). Current evidence suggested that diet and certain nutrients may influence sleep (6). Nutrients can act as an external zeitgeber to regulate our body's internal clock and hence affecting our sleep (7). One such nutrient is dietary protein (PRO). A low-PRO [<16% of energy (E%)] diet has been reported to be associated with poor sleep quality (4). However, on the contrary, results of consuming a high-PRO diet to improve sleep quality are inconsistent. A high-PRO diet was reported to decrease wake episodes and SL while it was also associated with difficulty in maintaining sleep (1, 4).

The research interest on the impact PRO consumption in sleep has been linked to the amino acid tryptophan (Trp). Trp is the precursor of the sleep regulating neurotransmitter serotonin and hormone melatonin (7). However, other than Trp, a high-PRO food source can increase the ingestion of the more abundant large neutral amino acids (LNAA), which have been reported to impede transportation of Trp through the blood brain barrier (BBB) (1). Current evidence suggested that Trp ingestion through our diet can improve sleep, but it is heavily dependent on its ability to cross the BBB (1). This could be one of the potential explanation of result inconsistencies in studies observing the impact of PRO on sleep. It has been suggested that a higher blood Trp:LNAA ratio may increase the brain uptake of Trp, which may aid the synthesis of serotonin to regulate sleep (7). One of the way to improve the bioavailability of Trp for this serotonergic pathway synthesis is by consuming PRO rich in Trp. Plant-based PRO are reported to be relatively high in Trp and has been associated with better sleep quality (6). However, a study by Zhou et al. has also reported that sleep, although favoring a higher PRO diet, was not influenced by the PRO source (8).

Currently, research on the association between PRO intake and sleep quality, especially in middle-aged and older adults, is limited. Additionally, there is still a lack of studies on the contribution of amino acid consumption and their sources to sleep quality. Hence, the aim of the current study is to investigate the association of PRO intake, their amino acid components, and sources with sleep quality in middle-aged and older adults residing in Singapore. In addition, the study analyzes the plasma amino acid concentrations of the recruited study subjects to examine their association with sleep quality.

## Materials and Methods

The Strengthening the Reporting of Observational Studies in Epidemiology (STROBE) statement was applied to guide the execution and reporting of this cross-sectional study (9). The STROBE checklist of this study can be accessed as part of the Supplementary Material.

### Study Design and Population

This cross-sectional study has been approved by the National Healthcare Group's Domain Specific Review Board (NHG DSRB) (reference number: 2018/00221) and was registered in ClinicalTrials.gov (reference number: NCT03554954). Informed consent was obtained from all the subjects and monetary compensation was provided for their participation.

The subjects were middle-aged and older adults residing in Singapore and were recruited between September 2018 and October 2019 based on the following inclusion criteria: (1) 50–75 years old; (2) ability to give informed consent; (3) no prior consumption of dietary supplementations, such as protein supplements and natural extracts, for the past 1 month; (4) no significant changes in their diet for the past year; and (5) have sufficient venous access for blood sampling.

On the day of the visit, data and samples were collected from the recruited subjects which included: (1) anthropometric measurements; (2) systolic and diastolic blood pressure; (3) dietary assessment; (4) sleep quality assessment; (5) perceived stress assessment; and (6) fasting blood.

A total of 108 subjects were recruited for this study. However, due to incomplete dietary data from 4 participants, data from 104 subjects were used for the final analysis.

### Anthropometric and Blood Pressure Measurements

The height and weight of the subjects were acquired using a stadiometer and digital scale *(Seca, Hamburg, Germany)*. The waist circumference was taken in accordance to the WHO guidelines, which is the mid-point between the lower margin of the last rib and the highest point of the hip bone (10). Blood pressure was measured using a portable blood pressure monitor *(Omron HEM-7121, Kyoto, Japan)*. All on-site measurements were done in a fasted state and in duplicates. Their average was then calculated as the final reading.

### Sleep Quality and Perceived Stress Assessment

Sleep quality data were evaluated using the Pittsburgh Sleep Quality Index (PSQI) (11). The PSQI consisted of 19 questions that involves 7 sleep components which are (1) subjective sleep quality; (2) SL; (3) sleep duration; (4) SE; (5) sleep disturbances; (6) the use of sleep medication; and (7) daytime dysfunction. The sum of these 7 components gives a global sleep score (GSS) between 0 and 21. A higher score indicates a poorer quality of sleep and individuals with GSS > 5 were clinically identified to have a poor sleep quality (11, 12). From the PSQI questionnaire, the following 4 main sleep components were extracted: (1) sleep duration (h); (2) GSS (a.u.); (3) SL (min); and (4) SE (%). The national sleep foundation (NSF) recommends a minimum of 7 h/day of sleep (13). A SE <85% and SL > 30 min on three or more nights in a week has also been defined to exceed the normal clinical threshold and may suggest the presence of insomnia (14).

In addition to the sleep quality assessment, the subjects' perceived stress was assessed. This was measured using a perceived stress scale (PSS), which consisted of 10 questions and each question contributed a maximum of 4 points. The sum of the points will yield a PSS score, where a higher score was associated with a higher perceived stress status (15).

All the sleep and stress questionnaires were answered based on the sleep and stress status of the subject for the past 1 month.

### Dietary Assessment

Dietary data were acquired using the 3-day food record (3DFR), 2 weekdays, and 1 weekend, and nutrient analysis was conducted using the Dietplan 7 software *(Forestfield Software Ltd, Horsham, SXW, UK)*. The U.S. Department of Agriculture (USDA) and the “Energy & Nutrient Composition of Food” database from the Health Promotion Board of Singapore was used as the main and secondary nutritional reference, respectively (16, 17). The PRO intake was analyzed in the form of percentage of energy intake (E%) from PRO. Additionally, dietary Trp (g) and the LNAA (g) composition were extracted. The LNAA consisted of valine (Val; g), isoleucine (Ile; g), leucine (Leu; g), tyrosine (Tyr; g), and phenylalanine (Phe; g). The Trp:LNAA ratio was then calculated from these extracted data. Missing amino acid composition data were cross-referenced and estimated using a close alternative from the USDA database (16). The total PRO intake and their amino acid contents were separated and further categorized into the following protein sources: (1) plant; (2) animal; and (3) dairy. Using the USDA database, the PRO classification of mixed food items, such as a cheeseburger, were further broken down into individual ingredient components where possible. The amount of each PRO classification was calculated proportionally based on the PRO content. Dietary magnesium (Mg), vitamin B6, B9 (folate), and B12 intakes were extracted since these nutrients are involved in the serotonin and melatonin synthesis pathway and deficiencies of these nutrients have been associated to sleep disruptions (18). All dietary data are reported as daily average consumption.

### Plasma Amino Acid Analysis

A 10-h fasting blood was collected from the antecubital vein by a phlebotomist into EDTA-treated vacutainers *(Greiner, USA)*. The collected blood was then centrifuged *(Eppendorf, Germany)* at 3,000 × *g*, 15 min at 4°C to yield blood plasma which were then aliquoted (500 μl), and stored at −80°C *(U725 Innova, Eppendorf, Germany)* for future analysis.

To determine plasma amino acid concentration, 10% sulfosalicylic acid (SSA) was added to the plasma sample (200 μl plasma + 20 μl 10% SSA) and incubated for 60 min at 4°C. The samples were then centrifuged (*Eppendorf, Germany)* at 12,000 × g, 15 min at 4°C. After deproteinization, the supernatants were filtered through a 0.22 μm ultrafiltration filter and re-centrifuged (12,000 × g, 15 min at 4°C). Samples were then stored at 4°C until they were ready to be analyzed.

A cation-exchange high performance liquid chromatography with post-column ninhydrin derivation *(ARACUS, membraPURE, Germany)* was used to analyze the free amino acid composition in the plasma samples. External amino acid standards were used to identify and quantify the plasma amino acids. From this amino acid analysis, plasma Trp (nmol/ml), LNAA (nmol/ml), and Trp:LNAA (nmol:nmol) were obtained. Chromatographic analysis of each sample was performed in duplicates.

### Power Calculation and Statistical Analysis

Previous research reported an inverse relationship between PRO E% and sleep quality (β = −0.24; *p* = 0.01) (19). Based on this result, a power calculation using G^*^Power 3.1 (Heinrich-Heine-Universität, Düsseldorf, Germany) was conducted. Assuming similar statistical association, a population size of ≥103 would yield ≥80% power at α = 0.05.

Sleep quality and nutrient intake comparison between two age groups (middle-aged vs. older adults) was performed *via* independent *t*-test. In this study, middle-aged adults are categorized as those <65 years, while older adults are those aged ≥65 years (20). The association between PRO and amino acids intakes with sleep quality components was examined *via* multiple linear regression (MLR). The following multivariate-adjusted associations were performed: (1) adjusted for age, gender, and BMI (Model 1); (2) Model 1 further adjusted for PSS (Model 2); (3) Model 2 further adjusted for Mg, vitamin B6, B9, and B12 (Model 3). The association among Mg, vitamin B6, B9, and B12, was also performed *via* MLR: (1) unadjusted (Model 0); (2) adjusted for age, gender, and BMI (Model 1); (3) Model 1 further adjusted for PSS (Model 2). Additionally, an independent *t*-test was performed to compare the mean values among PRO, amino acid intakes, and plasma amino acid of subjects based on their sleep quality status: (1) sleep duration <7 vs. ≥7 h; (2) GSS ≤ 5 and > 5 a.u.; (3) SL ≤ 30 and > 30 min; (4) SE ≤ 85 and > 85%. All statistical analysis were conducted using STATA/MP 13 (STATACORP LP, College station, TX, USA). All data were reported as mean ± SD and β-coefficient, where significance was accepted at *p* < 0.05.

### USDA Data Categorization

Additionally, to better understand the PRO composition of PRO sources, PRO and amino acids (Trp, Val, Ile, Leu, Tyr and Phe) data from the USDA nutrient database were extracted and manually categorized based on their PRO sources (16). The two main categories are plant and animal sourced PRO. Food items which contains both animal and plant, such as cheeseburger, were excluded. The foods classified into the plant PRO group were then further categorized into vegetables, fruits, grains, legumes, and nuts and seeds for plant PRO. Similarly, animal PRO was separated into red meat, poultry, fish and seafood, dairy, and eggs. Definition of these groups were based on the 2015–2020 Dietary Guidelines for Americans (21). Following the categorization, the PRO, Trp, LNAA, and Trp:LNAA contents of these groups were averaged and their SD obtained. To statistically evaluate how different the mean PRO and amino acid composition between PRO sources (plant vs. animal) and across the different PRO groups, and independent *t*-test and one-way ANOVA were performed, respectively. Similarly, this was conducted using STATA/MP 13 (STATACORP LP, College Station, TX, USA) and significance was accepted at *p* < 0.05.

## Results

### Baseline Characteristics

Table 1 summarizes the baseline characteristics of the 104 recruited subjects, where 43% (*n* = 45) are men and 57% (*n* = 59) are women. Recruited subjects have an average age of 59 ± 6 years old and are generally healthy. They have a mean GSS score of 5.0 ± 2.7 a.u., followed by a sleep duration, SL, SE, and PSS score of 6.5 ± 1.2 h, 16.0 ± 14.5 min, 90.4 ± 8.7%, and 12.1 ± 5.8 a.u., respectively.

**Table 1:** Baseline characteristics.

	**All (*****n*** **= 104)**		**Male (*****n*** **= 45)**		**Female (*****n*** **= 59)**	
	**Mean**	**SD**	**Mean**	**SD**	**Mean**	**SD**
Age (years)	59	6	61	6	57	6
BMI (kg/cm^2^)	23.9	4.2	24.7	4.9	23.2	0.5
Waist circumference (cm)	82.8	12.1	88.7	13.4	78.3	8.8
**Blood pressure**						
Systolic (mmHg)	116.8	17.1	122.8	16.0	112.3	16.6
Diastolic (mmHg)	74.9	10.7	78.8	10.6	71.9	9.8
**Sleep quality**						
Sleep duration (h)	6.5	1.2	6.5	1.3	6.5	1.1
GSS (a.u.)	5.0	2.7	4.8	2.7	5.2	2.8
Sleep latency (min)	16.0	14.5	16.3	13.9	15.8	15.2
Sleep efficiency (%)	90.4	8.7	91.2	8.1	89.8	9.2
Perceived stress (a.u.)	12.1	5.8	11.1	5.1	12.9	6.2

Table 2 compares the mean sleep quality components between subjects that are middle aged (<65 years; *n* = 91) and older (≥65 years; *n* = 13). In this study, older adults have a significantly longer sleep duration (7.1 ± 1.5 h) than those who are middle-aged (6.4 ± 1.1 h) (*p*: 0.049). No significant differences in GSS, SL, and SE were observed between the two age groups.

**Table 2:** Sleep quality and nutrient intake comparison between middle aged (<65 years) and older (≥65 years) adults.

	**Age < 65 years**		**Age ≥65 years**		***t*** **-test**
	**(*****n*** **= 91)**		**(*****n*** **= 13)**		
	**Mean**	**SD**	**Mean**	**SD**	***p*** **-value**
**Sleep quality**					
Sleep duration (h)	6.4	1.1	7.1	1.5	0.049*
GSS (a.u.)	5.2	2.6	3.7	3.3	0.066
Sleep latency (min)	16.7	15.0	11.0	9.7	0.185
Sleep efficiency (%)	90.2	8.8	91.7	8.4	0.567
Perceived stress (a.u.)	12.4	5.4	10.3	8.0	0.235
**Diet**					
PRO (E%)	18.7	4.2	18.4	3.2	0.824
Trp (g)	0.876	0.296	0.827	0.324	0.584
Trp:LNAA	0.047	0.003	0.047	0.006	0.633
Plant PRO (E%)	7.9	3.5	7.4	3.1	0.632
Plant Trp (g)	0.354	0.170	0.325	0.217	0.580
Plant Trp:LNAA	0.050	0.004	0.053	0.014	0.090
Animal PRO (E%)	10.3	4.0	10.6	2.6	0.834
Animal Trp (g)	0.520	0.232	0.500	0.175	0.775
Animal Trp:LNAA	0.045	0.003	0.044	0.002	0.415
Dairy PRO (E%)	0.8	1.0	0.7	0.8	0.579
Dairy Trp (g)	0.047	0.056	0.031	0.035	0.328
Dairy Trp:LNAA	0.038	0.022	0.032	0.019	0.426
Mg (mg)	313	143	269	101	0.283
Vitamin B6 (mg)	1.714	0.664	1.690	0.954	0.909
Vitamin B9 [Folate] (μg)	344	127	316	113	0.446
Vitamin B12 (μg)	3.27	2.23	5.69	9.93	0.044*
**Plasma amino acids**					
Trp (nmol/mL)	22.9	10.3	21.8	8.5	0.708
Trp:LNAA	0.077	0.014	0.076	0.014	0.932

* *p < 0.05*.

E%, percentage of energy intake; Mg, magnesium; PRO, dietary protein; Trp, tryptophan; Trp:LNAA, tryptophan: large neutral amino acid ratio.

### Nutrient Intakes and Plasma Amino Acids Concentration

Collected dietary data showed that subjects consumed an average of 18.7 ± 4.1 E% PRO daily (Table 3). In addition, it is tabulated that the PRO consumption was higher from animal-sourced PRO (10.4 ± 3.8 E%) as compared with plant-sourced PRO (7.9 ± 3.4 E%). Subjects consumed an average of 0.870 ± 0.298 g of Trp from their diet, where there was a mean Trp value of 0.351 ± 0.175 g obtained from plant sources and 0.517 ± 0.225 g from animal sources. There was a mean dietary Trp:LNAA consumption of 0.047 ± 0.003. Additionally, plant and animal protein sources consumed by the subjects averaged a Trp:LNAA value of 0.050 ± 0.006 and 0.045 ± 0.003, respectively. An average of 0.5 ± 0.8 E% of PRO was not able to be categorized into plant or animal sources. Other relevant nutrients intake, Mg, vitamin B6, B9, and B12 correspondingly had an average value of 307 ± 139 mg, 1.711 ± 0.701 mg, 341 ± 125 μg, and 3.57 ± 4.06 μg.

**Table 3:** Nutrient intakes and plasma amino acids concentration.

	**All (*****n*** **= 104)**		**Male (*****n*** **= 45)**		**Female (*****n*** **= 59)**	
	**Mean**	**SD**	**Mean**	**SD**	**Mean**	**SD**
**Diet**						
PRO (E%)	18.7	4.1	18.6	3.3	18.8	4.6
Trp (g)	0.870	0.298	0.917	0.332	0.835	0.267
Trp:LNAA	0.047	0.003	0.047	0.004	0.047	0.002
Plant PRO (E%)	7.9	3.4	7.8	2.7	7.9	3.9
Plant Trp (g)	0.351	0.175	0.365	0.169	0.339	0.181
Plant Trp:LNAA	0.050	0.006	0.050	0.008	0.050	0.004
Animal PRO (E%)	10.4	3.8	10.3	3.3	10.4	4.2
Animal Trp (g)	0.517	0.225	0.551	0.251	0.492	0.201
Animal Trp:LNAA	0.045	0.003	0.045	0.004	0.045	0.002
Dairy PRO (E%)	0.8	1.0	0.7	0.1	0.9	1.1
Dairy Trp (g)	0.045	0.054	0.049	0.063	0.042	0.046
Dairy Trp:LNAA	0.037	0.022	0.036	0.024	0.038	0.020
Uncategorized PRO (E%)	0.5	0.8	0.5	0.9	0.4	0.6
Mg (mg)	307	139	325	139	294	138
Vitamin B6 (mg)	1.711	0.701	1.781	0.710	1.657	0.695
Vitamin B9 [Folate] (μg)	341	125	366	128	321	121
Vitamin B12 (μg)	3.57	4.06	3.95	5.56	3.28	2.37
**Plasma amino acids**						
Trp (nmol/mL)	22.8	10.1	23.9	10.6	21.9	9.7
Trp:LNAA	0.077	0.014	0.075	0.015	0.078	0.014

*E%, percentage of energy intake; Mg, magnesium; PRO, dietary protein; Trp, tryptophan; Trp:LNAA, tryptophan: large neutral amino acid ratio*.

In this study, older adults were consuming a significantly higher amount of vitamin B12 (5.69 ± 9.93 μg) than those who are middle-aged (3.27 ± 2.23 μg) (Table 2; *p*: 0.044). No differences in other nutrient intakes were observed.

Additionally, average plasma Trp concentration and Trp:LNAA ratio were 22.8 ± 10.1 nmol/ml and 0.077 ± 0.014, respectively (Table 3). No differences in plasma Trp concentration and Trp:LNAA ratio were observed between middle-aged and older adults (Table 2).

### Association Between Nutrient Intake and Sleep Quality

Multiple linear regression was performed on PRO intake with sleep duration (Table 4). In Model 1, plant Trp:LNAA was found to be positively associated with sleep duration, while dairy Trp was negatively associated: plant Trp:LNAA (β_plant_: 43.857 h; *p*: 0.025); dairy Trp (β_dairy_: −4.961 h/g; *p*: 0.020). In Model 2, this association remained significant: plant Trp: LNAA (β_plant_: 43.986 h; *p*: 0.025); dairy Trp (β_dairy_: −4.980 h/g; *p*: 0.021) after PSS adjustment. After further adjustment for Mg, vitamin B6, B9, and B12 (Model 3), the association between sleep duration with plant Trp: LNAA (β_plant_: 54.006 h.; *p*: 0.008) and dairy Trp still remained (β_dairy_: −5.166 h/g; *p*: 0.017). Additionally, significant positive associations were observed for total Trp: LNAA (β_total_: 108.234 h; *p*: 0.005) and plant Trp (β_plant_: 2.653 h/g; *p*: 0.020) intake in Model 3. In Model 3, dairy PRO (β_dairy_: −23.646 h/%; *p*: 0.038) intake was negatively associated with sleep duration. Apart from sleep duration, GSS also had a positive association with animal PRO intake (Supplementary Table S1) in Model 3 (β_animal_: 17.316 a.u./E%; *p*: 0.034). Conversely, no significant association was observed for SL and SE% with any of the PRO intake (Supplementary Tables S2, S3).

**Table 4:** Association between sleep duration with dietary protein intakes.

	**Model 1**		**Model 2**		**Model 3**	
	**B**	***p*** **-value**	**β**	***p*** **-value**	**β**	***p*** **-value**
PRO (E%)	0.853	0.765	0.838	0.770	−1.304	0.659
Trp (g)	0.260	0.510	0.257	0.517	0.934	0.132
Trp:LNAA	71.929	0.051	72.428	0.051	108.234	0.005*
Plant PRO (E%)	−3.167	0.346	−3.175	0.347	−1.478	0.678
Plant Trp (g)	0.036	0.957	0.033	0.961	2.653	0.020*
Plant Trp:LNAA	43.857	0.025*	43.986	0.025*	54.006	0.008*
Animal PRO (E%)	3.667	0.223	3.652	0.228	0.496	0.882
Animal Trp (g)	0.411	0.429	0.408	0.436	0.143	0.822
Animal Trp:LNAA	3.969	0.920	4.349	0.913	12.287	0.748
Dairy PRO (E%)	−22.550	0.052	−22.531	0.054	−23.646	0.038*
Dairy Trp (g)	−4.961	0.020*	−4.980	0.021*	−5.166	0.017*
Dairy Trp:LNAA	−3.557	0.498	−3.506	0.508	0.030	0.996

*E%, percentage of energy intake; PRO, dietary protein; Trp, tryptophan; Trp:LNAA, tryptophan: large neutral amino acid ratio; LNAA (Val, Ile, Leu, Tyr, Phe)*.

*Model 1: Adjusted for age, gender, and BMI*.

*Model 2: Adjusted for age, gender, BMI, and PSS*.

Model 3: Adjusted for age, gender, BMI, PSS, Mg, Vitamin B6, B9, and B12. *p < 0.05.

The independent *t*-test comparison of PRO intake based on the subjects' sleep duration, GSS, SL, and SE status can be seen in Supplementary Tables S4–S7. It was found that subjects with a sleep duration ≥7 h consumed a lower amount of Trp from diary as compared with those with sleep duration <7 h (sleep duration ≥7 vs. <7 h: 0.029 ± 0.028 g vs. 0.057 ± 0.065 g; *p*: 0.009). Those with SE > 85% consumed significantly less PRO from diary sources as compared with those with SE ≤ 85% (SE > 85 vs. ≤ 85%: 0.7 ± 0.9 E% vs. 1.2 ± 1.3 E%; *p*: 0.033). No other significant differences were detected.

In addition to the PRO intake, a MLR analysis on Mg, vitamin B6, B9, and B12 was performed. After adjusting for age, gender, and BMI (Model 1), sleep duration was negatively associated with vitamin B9 (β_B9_: −0.002 h/μg; *p*: 0.039) (Supplementary Table S8). On the other hand, B9 was positively associated with SE (β_B9_: 0.014 h/%; *p*: 0.042). After adjusting Model 1 further for PSS level (Model 2), these associations remained.

### Association Between Plasma Amino Acids and Sleep Quality

An MLR analysis on plasma amino acids (Table 5) showed that sleep duration was negatively associated with plasma Trp: LNAA on all three multivariate MLR models: β_plasma_: −20.632 h; *p*: 0.012 (Model 1); β_plasma_: −20.609 h; *p*: 0.013 (Model 2); β_plasma_: −19.522 h; *p*: 0.014 (Model 3). In addition to the MLR analysis, a *t*-test comparison found that subjects with sleep duration ≥ 7 h has a lower plasma Trp:LNAA than those with sleep duration > 7 h (sleep duration ≥ 7 vs. <7 h: 0.072 ± 0.011 vs. 0.080 ± 0.015; *p*: 0.005) (Supplementary Table S4). However, this association was not observed with plasma Trp concentration for both MLR and *t*-test comparison.

**Table 5:** Association between sleep duration, global sleep score (GSS), sleep latency and sleep efficiency with plasma amino acids.

	**Model 1**		**Model 2**		**Model 3**	
	**β**	***p*** **-value**	**B**	***p*** **-value**	**β**	***p*** **-value**
**Sleep duration (h)**						
Trp (nmol/mL)	−0.005	0.691	−0.005	0.691	−0.007	0.534
Trp:LNAA	−20.632	0.012*	−20.609	0.013*	−19.522	0.014*
**GSS (a.u.)**						
Trp (nmol/mL)	0.013	0.638	0.011	0.663	0.013	0.633
Trp:LNAA	10.715	0.591	13.600	0.467	11.728	0.561
**Sleep latency (min)**						
Trp (nmol/mL)	0.268	0.065	0.264	0.067	0.269	0.067
Trp:LNAA	29.992	0.777	35.429	0.738	37.877	0.724
**Sleep efficiency (%)**						
Trp (nmol/mL)	−0.049	0.573	−0.047	0.587	−0.046	0.587
Trp:LNAA	30.365	0.629	27.382	0.663	21.766	0.726

*GSS, Global sleep score; Trp, tryptophan; Trp:LNAA, tryptophan: large neutral amino acid ratio*.

*Model 1: Adjusted for age, gender, and BMI*.

*Model 2: Adjusted for age, gender, BMI, and PSS*.

Model 3: Adjusted for age, gender, BMI, PSS, Mg, Vitamin B6, B9, and B12. *p < 0.05.

No statistically significant association was observed for GSS, SL, and SE% with plasma amino acids (Table 5).

### USDA Database Analysis

Animal source contained higher PRO, Trp, and LNAA as compared with plant source (PRO_animal_: 22.10 ± 7.42 g vs. PRO_plant_: 6.92 ± 8.69 g; Trp_animal_: 0.23 ± 0.10 g vs. Trp_plant_: 0.09 ± 0.12 g; LNAA_animal_: 5.56 ± 2.01 g vs. LNAA_plant_: 1.68 ± 2.23 g) (Supplementary Table S9). However, the plant source showed a higher Trp:LNAA ratio as compared with animal source (Trp:LNAA_animal_: 0.042 ± 0.009 vs. Trp:LNAA_plants_: 0.053 ± 0.024, *p*: < 0.001).

Supplementary Tables S10–S12, respectively show the PRO (g), Trp (g), and Trp:LNAA content comparison of different food protein sources (per 100 g). Results revealed that dairy has one of the lowest PRO, Trp, and Trp:LNAA profile as compared with other animal PRO sources (red meat, poultry, fish and seafood, and egg): PRO_dairy_: 11.2 ± 10.2 g; Trp_dairy_: 0.152 ± 0.150 g; Trp:LNAA_dairy_: 0.044 ± 0.015. On the other hand, egg protein has one of the highest PRO, Trp, and Trp:LNAA profile among the animal PRO: PRO_egg_: 26.8 ± 25.7 g; Trp_egg_: 0.401 ± 0.396 g; and Trp:LNAA_egg_: 0.049 ± 0.008. However, only the Trp content in egg is significantly higher than other animal PRO sources (*p* < 0.05).

Among the plants, nuts and seeds have the highest PRO and Trp content: PRO_nutsandseeds_: 16.5 ± 10.7 g; Trp_nutsandseeds_: 0.227 ± 0.174 g; and they also have one of the highest Trp:LNAA_nutsandseeds_: 0.053 ± 0.013. Compared with the other plant sources, fruits have the highest Trp:LNAA ratio: Trp:LNAA_fruits_: 0.061 ± 0.035. However, on the downside, they also have the lowest PRO and Trp (PRO_fruits_: 1.0 ± 1.2 g; Trp_fruits_: 0.009 ± 0.014 g).

## Discussion

Sleep is an integral part of our overall wellbeing as it has been shown to influence many biological metabolic (e.g., glucose regulation and inflammation) and psychological (e.g., memory and attention) processes. Consequently, poor sleep has been associated with the development of disease conditions (22). Therefore, developing strategies to improve the sleep is of importance, especially in older adults who are more susceptible to poorer sleep quality. Some studies have reported that nutrients, such as PRO, has certain benefits on sleep which includes promoting sleep induction and reducing night awakenings (3, 4). In this study, the association between dietary PRO and amino acids with sleep quality was examined in middle-aged and older Singaporean adults and we found an association between a higher total dietary Trp:LNAA intake with a better sleep duration. In addition, we observed that the source of the PRO may influence their association with sleep quality.

From the current study, dietary PRO (E%) and Trp intakes were not significantly associated with any of the sleep quality parameters. However, a significant positive association was observed for total dietary Trp: LNAA intake and sleep duration. The positive relationship of dietary Trp:LNAA on sleep duration has been suggested to be attributed to its influence on the bioavailability of plasma Trp for the BBB transport. Trp is an essential amino acid and can only be obtained externally from PRO in our diet or *via* supplementations (3). When Trp is consumed, it will be readily absorbed into the intestinal capillaries and transported to the brain *via* the blood (23). Circulating Trp in the blood will then be transported into the brain through the BBB, where it is converted to 5-hydroxytryptophan (5-HTP) by tryptophan 5-hydroxylase (TPH). 5-HTP will then undergo further enzymatic reaction to be converted to serotonin (3). However, LNAA competes with Trp for the BBB carrier transport (7). Compared with LNAA, the amount of Trp found in food is small, giving Trp a competitive disadvantage for BBB transport. This decreases its availability for serotonin synthesis (7, 24). Therefore, the uptake of Trp into the brain is dependent not only by the amount of Trp, but also a higher plasma Trp:LNAA ratio (7). Another important sleep-related compound produced from serotonin is the hormone melatonin. This hormone is produced in the pineal gland and plays an important role in regulating the body's circadian rhythm (25, 26). It has been reported that melatonin production decreases with age, especially in middle-aged and older individuals with insomnia. Melatonin synthesis in the pineal gland is dependent on the cytoplasmic availability of serotonin in the pinealocytes (27). The production of serotonin is restrained by the conversion of Trp to 5-HTP (28, 29). In the pineal gland, Trp is converted to 5-HTP by tryptophan 5-hydroxylase 1 (TPH1), a TPH isomer expressed in the pineal gland (30). Similar to TPH, TPH1 also has a low inherent affinity to Trp (28, 29). Hence, a higher plasma Trp:LNAA may facilitate the saturation and binding of TPH1 with Trp. This then may promote the production of serotonin in the gland and facilitate melatonin synthesis to promote sleep.

However, unexpectedly, plasma Trp:LNAA ratio showed a significant negative relationship with sleep duration in this study. A possible explanation of this observation would be that over time, due to a higher Trp:LNAA, more Trp could be transported into the brain and converted to the sleep-signaling neurotransmitter serotonin. This in turn may simultaneously decrease Trp: LNAA ratio in the plasma. Hence, analyzing the degree of the sleep-regulating hormone melatonin may help to confirm this speculation. This could be possibly done by analyzing urinary concentration of 6-sulfatoxymelatonin (6-aMTs), which is a metabolite of melatonin. Previous studies have found that, 6-aMTs rises following Trp supplementation (1).

Additionally, adjusting other relevant nutrients such as Mg, vitamin B6, B9, and B12 also provides an insight that the serotonergic pathway may be involved in explaining the association observed between total Trp:LNAA and sleep duration. Deficiencies of certain minerals and B vitamins have been reported to disrupt sleep and this association has been linked to their involvement in serotonin and melatonin synthesis (18). Mg, for example, has been found to increase the serotonin N-acetyltransferase activity which is one of the enzymes involved in the melatonin synthesis pathway (31). A randomized controlled trial (RCT) executed in an elderly population found that Mg supplementation was found to significantly improve the subjective and objective measures of sleep (32). Similarly, vitamin B12 has also been reported to enhance the secretion of nocturnal melatonin *via* its effect on the sleep-wake rhythm and this may explain the longer sleep duration in older adults observed in this study (33). In addition, vitamin B6 and B9 are known to influence serotonin and melatonin synthesis by acting as co-enzymes in the serotonergic pathways (34). However, more studies are required to confirm these speculations.

Unlike sleep duration and GSS, no association was observed between SL and SE with the PRO consumptions. This may be explained by the subject's sleep quality demographic. Subject's SL and SE are within the recommended range for good sleep which are ≤ 30 min and ≥85%, while sleep duration and GSS falls within the range that has been considered as poor sleep: sleep duration <7 h and GSS > 5 a.u. (11, 13, 14). It was reported that the effect of Trp may be more obviously seen in people with poorer sleep quality (35). Similar observations on the effect of Trp may be seen for the individual sleep quality components (35). As the majority of the subjects have a desirable SL and SE, it may be more difficult to detect the potential association between these sleep components and the PRO intakes. Hence, performing additional studies on the effect of PRO and dietary amino acid intakes on different sleep component in poor sleepers may provide a more discernable result.

Moreover, this study examined the association between sleep quality and intakes of PRO, Trp and Trp:LNAA from different sources. It was found that plant Trp and plant Trp:LNAA intakes are positively associated with sleep duration while this was not observed with animal sources. A comparison was made between the amino acid profile of plant and animal source PRO from the USDA database, and it showed that although animal food products contain higher PRO and Trp than plants, plant PRO has a higher Trp:LNAA ratio. Simultaneously, plants inherently contain dietary carbohydrate. Dietary carbohydrate has been reported to be able to increase plasma Trp:LNAA by 20–50% (36). Carbohydrate was reported to induce a selective insulin-mediated decrease of plasma LNAA hence, further improving the availability of Trp for BBB transport (29, 36). A similar result was observed in a preliminary cross-sectional study addressed in the review by St-Onge et al., where plant PRO intake was positively associated with better sleep quality and lower odds of short sleep duration (6). Other contributing factors which may provide some explanation are isoflavones and polyphenols that are rich in plant sources. Both compounds have been positively associated with sleep quality (37, 38). The biological mechanism to explain the relationship observed between isoflavone consumption and sleep has not been elucidated. However, it has been suggested that it may be linked to the possible effect of estrogen on serotonin (37, 39). Isoflavone may mimic estrogen and induce a serotonergic effect that modulate sleep. On the other hand, polyphenols may aid in sleep by acting as an anti-inflammatory agent (38). Research has reported that sleep disturbances are positively associated with inflammation and blood concentration of inflammatory markers, such interleukin-6 was higher in people with poorer sleep as compared with people with good sleep (40). However, clinical studies are still needed to confirm the effects of isoflavones and polyphenols on sleep quality. Lastly, higher plant PRO intake may indicate a higher intake of overall plants-based foods, such as fruits, vegetables, and wholegrains consumption. Increased consumption of fruits, vegetables, and wholegrains has been reported to support good sleep quality (4) and a better quality of diet which contains these foods showed a beneficial influence on sleep (6).

Other than looking just into plant vs. animal PRO, we further separated the animal PRO intake from dairy sources. One of the highest source of Trp which have a high Trp:LNAA ratio is α-lactalbumin (41). This is a whey-derived protein and its consumption has been found to increase Trp:LNAA ratio by 130% (42). Nevertheless, in this study, dairy PRO consumption was inversely associated with sleep duration. Previous studies have reported that α-lactalbumin demonstrated a favorable effect on sleep quality. However, the consumption of normal commercial milk had no effect on sleep (36, 43). One reason could be that dairy PRO, as a whole, contains not only α-lactalbumin, but also other whey and casein derived PRO (44). These proteins may inherently increase the LNAA content, and hence reduced the Trp:LNAA ratio. A comparison between different animal and plant PRO using USDA database, dairy PRO has the lowest PRO, Trp, and Trp:LNAA content within the animal PRO group. This in turn may also lower the availability of Trp for BBB transport. Another explanation is that the dairy consumption of the recruited participants is relatively low (PRO_dairy_: 0.8 E%) and their distribution within the study may be too narrow to properly execute a proper analysis.

One of the main weaknesses of this study is that the interpretation of collected data will only provide association relationship due to the nature of its cross-sectional study design. Thus, RCTs are required to confirm the causality nature of the observed results in this study. Additionally, although the study was carried out in Singapore, the intakes of amino acid and micronutrients were mainly estimated using USDA database due to the lack of available local database. As a result, there may be discrepancies between nutrient profiles in food found between these two countries. Additionally, although subjects have been instructed on how to standardize the quantification of their food intake, food record has been associated with under-reporting. This has been one of the main obstacle to accurately collect habitual dietary intake data (45). To improve the accuracy of the food record, in future studies, food consumed by the subjects can be quantified with the aid of an electronic food scale and photos. Other nutrients, such as vitamin D and zinc, have also been associated with sleep quality (3, 46). Evaluating their association and mechanistic involvement with sleep quality may give a more holistic understanding of diet and sleep. Another possible source of variation would be from the method of sleep data collection. Although a validated questionnaire was used, it is still a subjective measure of sleep and may not accurately reflect the subjects' actual sleep quality. To improve on this, an additional objective measure of sleep, such as the use of actigraphy watch or electroencephalogram will be helpful in improving the precision of the sleep data collection.

Nevertheless, the strengths of this study would be that it has been carried out in the aging Asian population. The data gathered also support established nutrition and sleep guidelines, especially in a similar population demographic. Another strength is the inclusion of plasma amino acid data since there are currently limited studies done which comprehensively examined dietary, plasma amino acid, and sleep as a package. In addition, we investigated an association between sleep quality components with different sources of PRO (plant vs. animal). Although it has not been further analyzed in this study, the USDA database analysis can be used to help identify potential PRO-rich food groups that may be beneficial for future sleep research. Most of the PRO and sleep research has been done with both plant and animal-sourced PRO together or α-lactalbumin. Other than just designing studies based on the total PRO intake or α-lactalbumin, we can also screen potential “sleep-inducing” food based on their Trp:LNAA ratio. For example, among the animal sourced PRO, eggs have the highest average PRO, Trp and Trp:LNAA. Egg protein hydrolysate has also been found to increase brain Trp availability (47). However, few trials assessed the impact of egg consumption on sleep quality. Additionally, another high Trp:LNAA food source is plant-sourced PRO, such as from nuts and seeds. RCTs assessing their influence on sleep can be potential research direction. This may be beneficial, as the current demand for plant-sourced protein is high and is expected to increase in the next decade (48). Lastly, previous sleep studies mostly focused on observing individual sleep component, such as sleep duration. In this study, we further assessed a wide range of sleep components, as sleep is a multidimensional concept (49).

## Conclusion

Sleep duration in middle-aged and older adults in Singapore is positively associated with dietary Trp and Trp:LNAA, in particular when obtained from plant sources. This suggest that the source of PRO, for example, plant PRO, may matter in its ability to improve sleep quality.

## Data Availability Statement

The original contributions presented in the study are included in the article/Supplementary Material, further inquiries can be directed to the corresponding author.

## Ethics Statement

The studies involving human participants were reviewed and approved by National Healthcare Group's Domain Specific Review Board (NHG DSRB) (reference number: 2018/00221). The participants provided their written informed consent to participate in this study.

## Author Contributions

CS and JK designed the study, drafted/revised the manuscript, and have primary responsibility for final content. CS, WL, DT, and DL conducted the study. CS and WL performed data extraction. CS performed the analysis and interpretation of the data. All authors read and approved the final version of the manuscript.

## Funding

This research was funded by the National University of Singapore (Grant number: R-160-000-A03-133) and the NUS iHealthtech Microbiome in Health, Disease and Aging (Grant number: R-160-000-A89-133).

## Conflict of Interest

The authors declare that the research was conducted in the absence of any commercial or financial relationships that could be construed as a potential conflict of interest.

## Publisher's Note

All claims expressed in this article are solely those of the authors and do not necessarily represent those of their affiliated organizations, or those of the publisher, the editors and the reviewers. Any product that may be evaluated in this article, or claim that may be made by its manufacturer, is not guaranteed or endorsed by the publisher.
