# Supplementary material for: Association Between Dietary Protein Intake and Sleep Quality in Middle-Aged and Older Adults in Singapore
Source: Front Nutr. 2022 Mar 9;9:832341. doi: 10.3389/fnut.2022.832341 (PMC8959711; doi:10.3389/fnut.2022.832341)
Supplement: Supplementary file 1 [file Table_1.DOCX]

**Table S1.** Association between GSS with dietary protein intakes

|  | **Model 1** | | **Model 2** | | **Model 3** | | |  |
| --- | --- | --- | --- | --- | --- | --- | --- | --- |
|  | **β** | **p-value** | **β** | **p-value** | **β** | **p-value** | |  |
| PRO (E%) | 7.381 | 0.277 | 6.561 | 0.303 | 10.582 | 0.149 | |  |
| Trp (g) | -0.275 | 0.771 | -0.462 | 0.602 | 0.986 | 0.525 | |  |
| Trp:LNAA | -88.369 | 0.318 | -72.672 | 0.382 | -107.843 | 0.266 | |  |
| Plant PRO (E%) | -1.387 | 0.863 | -1.731 | 0.818 | -1.771 | 0.842 | |  |
| Plant Trp (g) | -1.262 | 0.424 | -1.428 | 0.335 | -2.512 | 0.381 | |  |
| Plant Trp:LNAA | 9.153 | 0.847 | 13.904 | 0.754 | 9.322 | 0.855 | |  |
| Animal PRO (E%) | 10.971 | 0.126 | 9.951 | 0.140 | 17.316 | 0.034* | |  |
| Animal Trp (g) | 0.279 | 0.823 | 0.062 | 0.958 | 1.733 | 0.269 | |  |
| Animal Trp:LNAA | -85.805 | 0.360 | -67.561 | 0.443 | -80.639 | 0.396 | |  |
| Dairy PRO (E%) | 36.813 | 0.186 | 37.938 | 0.146 | 40.663 | 0.155 | |  |
| Dairy Trp (g) | 6.321 | 0.220 | 5.667 | 0.242 | 7.479 | 0.170 | |  |
| Dairy Trp:LNAA | 5.708 | 0.648 | 9.809 | 0.405 | 10.152 | 0.450 | |  |
| *p-value <0.05  *Abbreviations:* E% (percentage of energy intake); GSS (Global Sleep Score); Mg (magnesium); PRO (dietary protein); Trp (tryptophan);Trp:LNAA (tryptophan: large neutral amino acid ratio); LNAA (Val, Ile, Leu, Tyr, Phe) | | | | | | | | |
| *Model 1: Adjusted for age, gender and BMI* | | | | | | |  |  |
| *Model 2: Adjusted for age, gender, BMI and PSS* | | | | | | |  |  |
| *Model 3: Adjusted for age, gender, BMI, PSS, Mg, Vitamin B6, B9 and B12* | | | | | | |  |  |
