# Supplementary material for: Association Between Dietary Protein Intake and Sleep Quality in Middle-Aged and Older Adults in Singapore
Source: Front Nutr. 2022 Mar 9;9:832341. doi: 10.3389/fnut.2022.832341 (PMC8959711; doi:10.3389/fnut.2022.832341)
Supplement: Supplementary file 2 [file Table_2.DOCX]

**Table S2.** Association between sleep latency with dietary protein intakes

|  | **Model 1** | | **Model 2** | | **Model 3** | | |  |
| --- | --- | --- | --- | --- | --- | --- | --- | --- |
|  | **β** | **p-value** | **β** | **p-value** | **β** | **p-value** | |  |
| PRO (E%) | -22.056 | 0.543 | -23.639 | 0.513 | -11.353 | 0.772 | |  |
| Trp (g) | -2.151 | 0.668 | -2.507 | 0.617 | 1.929 | 0.815 | |  |
| Trp:LNAA | -190.258 | 0.687 | -160.828 | 0.733 | -156.006 | 0.764 | |  |
| Plant PRO (E%) | -30.580 | 0.474 | -31.230 | 0.463 | -21.627 | 0.646 | |  |
| Plant Trp (g) | -5.213 | 0.536 | -5.527 | 0.510 | -6.504 | 0.671 | |  |
| Plant Trp:LNAA | 119.736 | 0.634 | 128.743 | 0.608 | 158.602 | 0.561 | |  |
| Animal PRO (E%) | 10.283 | 0.789 | 8.349 | 0.827 | 17.815 | 0.687 | |  |
| Animal Trp (g) | -0.478 | 0.942 | -0.888 | 0.893 | 4.059 | 0.629 | |  |
| Animal Trp:LNAA | -251.593 | 0.614 | -217.591 | 0.663 | -210.281 | 0.678 | |  |
| Dairy PRO (E%) | 47.373 | 0.750 | 49.484 | 0.739 | 85.803 | 0.574 | |  |
| Dairy Trp (g) | 6.488 | 0.814 | 5.252 | 0.848 | 16.112 | 0.580 | |  |
| Dairy Trp:LNAA | -14.431 | 0.829 | -6.934 | 0.917 | 8.521 | 0.906 | |  |
| *Abbreviations:* E% (percentage of energy intake); PRO (dietary protein); Trp (tryptophan);Trp:LNAA (tryptophan: large neutral amino acid ratio); LNAA (Val, Ile, Leu, Tyr, Phe) | | | | | | | | |
| *Model 1: Adjusted for age, gender and BMI* | | | | | | |  |  |
| *Model 2: Adjusted for age, gender, BMI and PSS* | | | | | | |  |  |
| *Model 3: Adjusted for age, gender, BMI, PSS, Mg, Vitamin B6, B9 and B12* | | | | | | |  |  |
|  | | | | | | |  |  |
