# Supplementary material for: Association Between Dietary Protein Intake and Sleep Quality in Middle-Aged and Older Adults in Singapore
Source: Front Nutr. 2022 Mar 9;9:832341. doi: 10.3389/fnut.2022.832341 (PMC8959711; doi:10.3389/fnut.2022.832341)
Supplement: Supplementary file 3 [file Table_3.DOCX]

**Table S3.** Association between sleep efficiency with dietary protein intakes

|  | **Model 1** | | **Model 2** | | **Model 3** | | |  |
| --- | --- | --- | --- | --- | --- | --- | --- | --- |
|  | **β** | **p-value** | **β** | **p-value** | **β** | **p-value** | |  |
| PRO (E%) | -0.438 | 0.984 | 0.437 | 0.984 | -0.910 | 0.968 | |  |
| Trp (g) | 3.588 | 0.226 | 3.795 | 0.200 | -0.405 | 0.932 | |  |
| Trp:LNAA | -35.824 | 0.898 | -52.757 | 0.850 | -191.605 | 0.524 | |  |
| Plant PRO (E%) | 23.499 | 0.353 | 23.865 | 0.344 | 18.399 | 0.499 | |  |
| Plant Trp (g) | 6.170 | 0.215 | 6.350 | 0.201 | -0.829 | 0.925 | |  |
| Plant Trp:LNAA | -198.083 | 0.183 | -203.258 | 0.171 | -280.345 | 0.073 | |  |
| Animal PRO (E%) | -19.195 | 0.398 | -18.128 | 0.424 | -20.708 | 0.417 | |  |
| Animal Trp (g) | 2.530 | 0.519 | 2.766 | 0.480 | -0.037 | 0.994 | |  |
| Animal Trp:LNAA | 220.929 | 0.455 | 202.031 | 0.495 | 150.868 | 0.606 | |  |
| Dairy PRO (E%) | -70.205 | 0.426 | -71.399 | 0.417 | -83.693 | 0.342 | |  |
| Dairy Trp (g) | -4.766 | 0.770 | -4.072 | 0.803 | -8.540 | 0.611 | |  |
| Dairy Trp:LNAA | -18.017 | 0.648 | -22.453 | 0.571 | -56.560 | 0.172 | |  |
| *Abbreviations:* E% (percentage of energy intake); PRO (dietary protein); Trp (tryptophan);Trp:LNAA (tryptophan: large neutral amino acid ratio); LNAA (Val, Ile, Leu, Tyr, Phe) | | | | | | | | |
| *Model 1: Adjusted for age, gender and BMI* | | | | | | |  |  |
| *Model 2: Adjusted for age, gender, BMI and PSS* | | | | | | |  |  |
| *Model 3: Adjusted for age, gender, BMI, PSS, Mg, Vitamin B6, B9 and B12* | | | | | | |  |  |
