# Supplementary material for: Association Between Dietary Protein Intake and Sleep Quality in Middle-Aged and Older Adults in Singapore
Source: Front Nutr. 2022 Mar 9;9:832341. doi: 10.3389/fnut.2022.832341 (PMC8959711; doi:10.3389/fnut.2022.832341)
Supplement: Supplementary file 4 [file Table_4.docx]

**Table S4.** Nutrient intakes and plasma amino acids concentration comparison between subjects with sleep duration < 7 h and ≥ 7 h.

|  |  | **Sleep Duration < 7 h**  **(n=60)** | |  | **Sleep Duration ≥ 7 h**  **(n=44)** | | **t-test** |
| --- | --- | --- | --- | --- | --- | --- | --- |
|  |  | **Mean** | **SD** |  | **Mean** | **SD** | **p-value** |
| **Diet** |  |  | |  |  | |  |
| PRO (E%) |  | 18.6 | 4.6 |  | 18.8 | 3.2 | 0.816 |
| Trp (g) |  | 0.880 | 0.302 |  | 0.857 | 0.295 | 0.697 |
| Trp:LNAA |  | 0.047 | 0.003 |  | 0.047 | 0.004 | 0.922 |
| Plant PRO (E%) |  | 8.3 | 4.0 |  | 7.2 | 2.2 | 0.099 |
| Plant Trp (g) |  | 0.376 | 0.187 |  | 0.316 | 0.154 | 0.082 |
| Plant Trp:LNAA |  | 0.050 | 0.004 |  | 0.050 | 0.008 | 0.920 |
| Animal PRO (E%) |  | 9.8 | 3.7 |  | 11.1 | 3.8 | 0.077 |
| Animal Trp (g) |  | 0.502 | 0.225 |  | 0.537 | 0.226 | 0.436 |
| Animal Trp:LNAA |  | 0.045 | 0.004 |  | 0.045 | 0.002 | 0.500 |
| Dairy PRO (E%) |  | 1.0 | 1.1 |  | 0.6 | 0.7 | 0.062 |
| Dairy Trp (g) |  | 0.057 | 0.065 |  | 0.029 | 0.028 | 0.009* |
| Dairy Trp:LNAA |  | 0.037 | 0.021 |  | 0.036 | 0.023 | 0.785 |
| Mg (mg) |  | 328 | 157 |  | 279 | 104 | 0.070 |
| Vitamin B6 (mg) |  | 1.738 | 0.642 |  | 1.674 | 0.781 | 0.650 |
| Vitamin B9 [Folate] (µg) |  | 356 | 124 |  | 319 | 126 | 0.143 |
| Vitamin B12 (µg) |  | 3.26 | 2.02 |  | 3.98 | 5.79 | 0.374 |
| **Plasma Amino Acids** |  |  | |  |  | |  |
| Trp (nmol/mL) |  | 23.4 | 10.8 |  | 21.9 | 9.0 | 0.440 |
| Trp:LNAA |  | 0.080 | 0.015 |  | 0.072 | 0.011 | 0.005* |
| *p-value <0.05  *Abbreviations:* E% (percentage of energy intake); Mg (magnesium); PRO (dietary protein); Trp (tryptophan); Trp:LNAA (tryptophan: large neutral amino acid ratio) | | | | | | | |
