# Supplementary material for: Association Between Dietary Protein Intake and Sleep Quality in Middle-Aged and Older Adults in Singapore
Source: Front Nutr. 2022 Mar 9;9:832341. doi: 10.3389/fnut.2022.832341 (PMC8959711; doi:10.3389/fnut.2022.832341)
Supplement: Supplementary file 5 [file Table_5.DOCX]

**Table S5.** Nutrient intakes and plasma amino acids concentration comparison between subjects with GSS ≤ 5 a.u. and > 5 a.u..

|  |  | **GSS ≤ 5 a.u.**  **(n=66)** | |  | **GSS > 5 a.u.**  **(n=38)** | | **t-test** |
| --- | --- | --- | --- | --- | --- | --- | --- |
|  |  | **Mean** | **SD** |  | **Mean** | **SD** | **p-value** |
| **Diet** |  |  | |  |  | |  |
| PRO (E%) |  | 18.3 | 4.0 |  | 19.3 | 4.2 | 0.259 |
| Trp (g) |  | 0.874 | 0.302 |  | 0.864 | 0.294 | 0.870 |
| Trp:LNAA |  | 0.047 | 0.004 |  | 0.047 | 0.002 | 0.367 |
| Plant PRO (E%) |  | 8.0 | 4.0 |  | 7.6 | 2.2 | 0.544 |
| Plant Trp (g) |  | 0.357 | 0.179 |  | 0.340 | 0.171 | 0.648 |
| Plant Trp:LNAA |  | 0.051 | 0.007 |  | 0.050 | 0.004 | 0.728 |
| Animal PRO (E%) |  | 9.9 | 3.7 |  | 11.2 | 4.0 | 0.080 |
| Animal Trp (g) |  | 0.516 | 0.239 |  | 0.520 | 0.201 | 0.930 |
| Animal Trp:LNAA |  | 0.045 | 0.003 |  | 0.045 | 0.002 | 0.750 |
| Dairy PRO (E%) |  | 0.7 | 0.9 |  | 1.1 | 1.2 | 0.056 |
| Dairy Trp (g) |  | 0.041 | 0.049 |  | 0.053 | 0.062 | 0.274 |
| Dairy Trp:LNAA |  | 0.036 | 0.022 |  | 0.038 | 0.022 | 0.683 |
| Mg (mg) |  | 306 | 126 |  | 309 | 161 | 0.923 |
| Vitamin B6 (mg) |  | 1.752 | 0.745 |  | 1.638 | 0.621 | 0.427 |
| Vitamin B9 [Folate] (µg) |  | 347 | 125 |  | 329 | 127 | 0.482 |
| Vitamin B12 (µg) |  | 3.76 | 4.92 |  | 3.23 | 1.77 | 0.526 |
| **Plasma Amino Acids** |  |  | |  |  | |  |
| Trp (nmol/mL) |  | 21.7 | 9.2 |  | 24.6 | 11.4 | 0.161 |
| Trp:LNAA |  | 0.075 | 0.013 |  | 0.079 | 0.016 | 0.161 |
| *Abbreviations:* E% (percentage of energy intake); Mg (magnesium); PRO (dietary protein); Trp (tryptophan); Trp:LNAA (tryptophan: large neutral amino acid ratio) | | | | | | | |
