# Supplementary material for: Association Between Dietary Protein Intake and Sleep Quality in Middle-Aged and Older Adults in Singapore
Source: Front Nutr. 2022 Mar 9;9:832341. doi: 10.3389/fnut.2022.832341 (PMC8959711; doi:10.3389/fnut.2022.832341)
Supplement: Supplementary file 6 [file Table_6.docx]

**Table S6.** Nutrient intakes and plasma amino acids concentration comparison between subjects with sleep latency ≤ 30 min and > 30 min.

|  |  | **SL ≤ 30 min**  **(n=94)** | |  | **SL ≤ 30 min**  **(n=94)** | | **t-test** |
| --- | --- | --- | --- | --- | --- | --- | --- |
|  |  | **Mean** | **SD** |  | **Mean** | **SD** | **p-value** |
| **Diet** |  |  | |  |  | |  |
| PRO (E%) |  | 18.7 | 4.2 |  | 18.3 | 2.9 | 0.780 |
| Trp (g) |  | 0.871 | 0.305 |  | 0.857 | 0.237 | 0.889 |
| Trp:LNAA |  | 0.047 | 0.003 |  | 0.046 | 0.002 | 0.450 |
| Plant PRO (E%) |  | 7.9 | 3.6 |  | 7.4 | 1.7 | 0.673 |
| Plant Trp (g) |  | 0.353 | 0.183 |  | 0.330 | 0.082 | 0.703 |
| Plant Trp:LNAA |  | 0.050 | 0.006 |  | 0.050 | 0.004 | 0.789 |
| Animal PRO (E%) |  | 10.3 | 3.9 |  | 10.6 | 3.7 | 0.812 |
| Animal Trp (g) |  | 0.516 | 0.228 |  | 0.525 | 0.202 | 0.911 |
| Animal Trp:LNAA |  | 0.045 | 0.003 |  | 0.045 | 0.003 | 0.748 |
| Dairy PRO (E%) |  | 0.8 | 1.0 |  | 0.6 | 0.5 | 0.529 |
| Dairy Trp (g) |  | 0.046 | 0.056 |  | 0.033 | 0.031 | 0.470 |
| Dairy Trp:LNAA |  | 0.036 | 0.022 |  | 0.044 | 0.024 | 0.290 |
| Mg (mg) |  | 309 | 142 |  | 296 | 103 | 0.784 |
| Vitamin B6 (mg) |  | 1.720 | 0.731 |  | 1.624 | 0.313 | 0.682 |
| Vitamin B9 [Folate] (µg) |  | 342 | 129 |  | 325 | 88 | 0.687 |
| Vitamin B12 (µg) |  | 3.66 | 4.24 |  | 2.73 | 1.35 | 0.497 |
| **Plasma Amino Acids** |  |  | |  |  | |  |
| Trp (nmol/mL) |  | 22.5 | 9.7 |  | 25.8 | 13.1 | 0.318 |
| Trp:LNAA |  | 0.076 | 0.014 |  | 0.079 | 0.016 | 0.634 |
| *Abbreviations:* E% (percentage of energy intake); Mg (magnesium); PRO (dietary protein); Trp (tryptophan); Trp:LNAA (tryptophan: large neutral amino acid ratio) | | | | | | | |
