# Supplementary material for: Association Between Dietary Protein Intake and Sleep Quality in Middle-Aged and Older Adults in Singapore
Source: Front Nutr. 2022 Mar 9;9:832341. doi: 10.3389/fnut.2022.832341 (PMC8959711; doi:10.3389/fnut.2022.832341)
Supplement: Supplementary file 7 [file Table_7.DOCX]

**Table S7.** Nutrient intakes and plasma amino acids concentration comparison between subjects with sleep efficiency ≤ 85% and > 85%.

|  |  | **SE ≤ 85%**  **(n=24)** | |  | **SE > 85%**  **(n=80)** | | **t-test** |
| --- | --- | --- | --- | --- | --- | --- | --- |
|  |  | **Mean** | **SD** |  | **Mean** | **SD** | **p-value** |
| **Diet** |  |  | |  |  | |  |
| PRO (E%) |  | 19.0 | 3.0 |  | 18.6 | 4.3 | 0.666 |
| Trp (g) |  | 0.873 | 0.283 |  | 0.869 | 0.304 | 0.952 |
| Trp:LNAA |  | 0.047 | 0.005 |  | 0.047 | 0.003 | 0.581 |
| Plant PRO (E%) |  | 7.1 | 1.4 |  | 8.1 | 3.8 | 0.191 |
| Plant Trp (g) |  | 0.339 | 0.169 |  | 0.354 | 0.178 | 0.712 |
| Plant Trp:LNAA |  | 0.052 | 0.010 |  | 0.050 | 0.004 | 0.098 |
| Animal PRO (E%) |  | 11.4 | 3.4 |  | 10.0 | 3.9 | 0.112 |
| Animal Trp (g) |  | 0.530 | 0.180 |  | 0.513 | 0.237 | 0.747 |
| Animal Trp:LNAA |  | 0.045 | 0.003 |  | 0.045 | 0.003 | 0.744 |
| Dairy PRO (E%) |  | 1.2 | 1.3 |  | 0.7 | 0.9 | 0.033* |
| Dairy Trp (g) |  | 0.057 | 0.061 |  | 0.041 | 0.051 | 0.217 |
| Dairy Trp:LNAA |  | 0.042 | 0.022 |  | 0.035 | 0.022 | 0.180 |
| Mg (mg) |  | 297 | 130 |  | 310 | 142 | 0.682 |
| Vitamin B6 (mg) |  | 1.621 | 0.413 |  | 1.738 | 0.767 | 0.478 |
| Vitamin B9 [Folate] (µg) |  | 319 | 108 |  | 347 | 130 | 0.345 |
| Vitamin B12 (µg) |  | 3.51 | 2.05 |  | 3.59 | 4.50 | 0.933 |
| **Plasma Amino Acids** |  |  | |  |  | |  |
| Trp (nmol/mL) |  | 23.4 | 10.3 |  | 22.6 | 10.1 | 0.731 |
| Trp:LNAA |  | 0.078 | 0.017 |  | 0.076 | 0.013 | 0.566 |
| *p-value <0.05  *Abbreviations:* E% (percentage of energy intake); Mg (magnesium); PRO (dietary protein); Trp (tryptophan); Trp:LNAA (tryptophan: large neutral amino acid ratio) | | | | | | | |
