# Supplementary material for: Association Between Dietary Protein Intake and Sleep Quality in Middle-Aged and Older Adults in Singapore
Source: Front Nutr. 2022 Mar 9;9:832341. doi: 10.3389/fnut.2022.832341 (PMC8959711; doi:10.3389/fnut.2022.832341)
Supplement: Supplementary file 8 [file Table_8.docx]

**Table S8.** Association between sleep duration, GSS, sleep latency and sleep efficiency with dietary Mg, vitamin B6, B9 and B12.

| **Sleep Duration (h)** | | | | | | |
| --- | --- | --- | --- | --- | --- | --- |
|  | ***Model 0*** | | ***Model 1*** | | ***Model 2*** | |
|  | **β** | **p-value** | **β** | **p-value** | **β** | **p-value** |
| Mg (mg) | -0.001 | 0.311 | -0.001 | 0.310 | -0.001 | 0.300 |
| Vitamin B6 (mg) | 0.064 | 0.697 | 0.046 | 0.780 | 0.047 | 0.780 |
| Vitamin B9 [Folate] (ug) | -0.002 | 0.042* | -0.002 | 0.039* | -0.002 | 0.040* |
| Vitamin B12 (ug) | 0.044 | 0.123 | 0.038 | 0.221 | 0.038 | 0.225 |
| **GSS (a.u.)** | | | | | | |
|  | ***Model 0*** | | ***Model 1*** | | ***Model 2*** | |
|  | **β** | **p-value** | **β** | **p-value** | **β** | **p-value** |
| Mg (mg) | -0.001 | 0.730 | -0.001 | 0.784 | -0.001 | 0.468 |
| Vitamin B6 (mg) | -0.444 | 0.251 | -0.414 | 0.294 | -0.408 | 0.271 |
| Vitamin B9 [Folate] (ug) | -0.002 | 0.350 | -0.002 | 0.401 | -0.002 | 0.443 |
| Vitamin B12 (ug) | -0.030 | 0.658 | -0.020 | 0.790 | -0.024 | 0.727 |
| **Sleep Latency (min)** | | | | | | |
|  | ***Model 0*** | | ***Model 1*** | | ***Model 2*** | |
|  | **β** | **p-value** | **β** | **p-value** | **β** | **p-value** |
| Mg (mg) | -0.006 | 0.574 | -0.006 | 0.557 | -0.008 | 0.462 |
| Vitamin B6 (mg) | -1.593 | 0.439 | -1.620 | 0.441 | -1.609 | 0.443 |
| Vitamin B9 [Folate] (ug) | -0.007 | 0.522 | -0.008 | 0.488 | -0.008 | 0.513 |
| Vitamin B12 (ug) | -0.415 | 0.242 | -0.460 | 0.239 | -0.469 | 0.229 |
| **Sleep Efficiency (%)** | | | | | | |
|  | ***Model 0*** | | ***Model 1*** | | ***Model 2*** | |
|  | **β** | **p-value** | **β** | **p-value** | **β** | **p-value** |
| Mg (mg) | 0.007 | 0.293 | 0.007 | 0.261 | 0.008 | 0.204 |
| Vitamin B6 (mg) | 1.954 | 0.112 | 2.012 | 0.105 | 2.005 | 0.105 |
| Vitamin B9 [Folate] (ug) | 0.013 | 0.057 | 0.014 | 0.042* | 0.014 | 0.046* |
| Vitamin B12 (ug) | -0.018 | 0.931 | -0.124 | 0.593 | -0.119 | 0.607 |
| *p-value <0.05  *Abbreviations: GSS (Global sleep score);* Trp (tryptophan);Trp:LNAA (tryptophan: large neutral amino acid ratio) | | | | | | |
| *Model 0: Unadjusted*  *Model 1: Adjusted for age, gender and BMI* | | | | | | |
| *Model 2: Adjusted for age, gender, BMI and PSS* | | | | | | |
