# Supplementary material for: Association Between Dietary Protein Intake and Sleep Quality in Middle-Aged and Older Adults in Singapore
Source: Front Nutr. 2022 Mar 9;9:832341. doi: 10.3389/fnut.2022.832341 (PMC8959711; doi:10.3389/fnut.2022.832341)
Supplement: Supplementary file 9 [file Table_9.docx]

**Table S9.** Comparison of protein and amino acids between animal source and plant source (per 100g) in USDA database

|  | **Protein Source (per 100g)** | | | | **t-test** |
| --- | --- | --- | --- | --- | --- |
|  | **Animal** | | **Plant** | |  |
|  | **Mean** | **SD** | **Mean** | **SD** | **p-value** |
| Total protein (g) | 22.10 | 7.42 | 6.92 | 8.69 | <0.001 |
| Trp (g) | 0.23 | 0.10 | 0.09 | 0.12 |  |
| LNAA (g) | 5.56 | 2.01 | 1.68 | 2.23 |  |
| Trp:LNAA | 0.042 | 0.009 | 0.053 | 0.024 |  |
| *Abbreviations:* Trp (tryptophan); Trp:LNAA (tryptophan: large neutral amino acid ratio); LNAA (Val, Ile, Leu, Tyr, Phe) | | | | | |
