# Supplementary material for: Association Between Dietary Protein Intake and Sleep Quality in Middle-Aged and Older Adults in Singapore
Source: Front Nutr. 2022 Mar 9;9:832341. doi: 10.3389/fnut.2022.832341 (PMC8959711; doi:10.3389/fnut.2022.832341)
Supplement: Supplementary file 10 [file Table_10.docx]

**Table S10.** Comparison of protein (g) content between different animal and plant-sourced proteins groups (per 100g) in USDA database.

| **Protein (g)** | **Mean** | **SD** | **Value Differences between Protein Sources** | | | | | | | | | | |
| --- | --- | --- | --- | --- | --- | --- | --- | --- | --- | --- | --- | --- | --- |
|  |  |  | **Red Meat** | **Poultry** | **Fish Seafood** | **Dairy** | **Eggs** | **Vegetables** | **Fruits** | **Grains** | **Legumes** | **Nuts and Seeds** |  |
| **Red Meat** | 23.5 | 5.8 |  |  |  |  |  |  |  |  |  |  |  |
| **Poultry** | 22.2 | 5.5 | -1.24* |  |  |  |  |  |  |  |  |  |  |
| **Fish and Seafood** | 20.4 | 5.8 | -3.10* | -1.86* |  |  |  |  |  |  |  |  |  |
| **Dairy** | 11.2 | 10.2 | -12.22* | -10.97* | -9.11* |  |  |  |  |  |  |  |  |
| **Eggs** | 26.8 | 25.7 | 3.35 | 4.59* | 6.45* | 15.57* |  |  |  |  |  |  |  |
| **Vegetables** | 3.0 | 3.9 | -20.42* | -19.17* | -17.31* | -8.20* | -23.77* |  |  |  |  |  |  |
| **Fruits** | 1.0 | 1.2 | -22.51* | -21.27* | -19.41* | -10.30* | -25.86* | -2.10* |  |  |  |  |  |
| **Grains** | 8.7 | 3.9 | -14.79* | -13.54* | -11.69* | -2.57* | -18.14* | 5.63* | 7.72* |  |  |  |  |
| **Legumes** | 13.6 | 13.7 | -9.83* | -8.59* | -6.73* | 2.38* | -13.18* | 10.58* | 12.68* | 4.96* |  |  |  |
| **Nuts and Seeds** | 16.5 | 10.7 | -6.96* | -5.71* | -3.85* | 5.26* | -10.31* | 13.46* | 15.55* | 7.83* | 2.88* |  |  |

*p-value <0.05
