# Supplementary material for: Association Between Dietary Protein Intake and Sleep Quality in Middle-Aged and Older Adults in Singapore
Source: Front Nutr. 2022 Mar 9;9:832341. doi: 10.3389/fnut.2022.832341 (PMC8959711; doi:10.3389/fnut.2022.832341)
Supplement: Supplementary file 11 [file Table_11.docx]

**Table S11.** Comparison of tryptophan (g) content between different animal and plant-sourced proteins groups (per 100g) in USDA database

| **Trp (g)** | **Mean** | **SD** | **Value Differences between Protein Sources** | | | | | | | | | |
| --- | --- | --- | --- | --- | --- | --- | --- | --- | --- | --- | --- | --- |
|  |  |  | **Red Meat** | **Poultry** | **Fish Seafood** | **Dairy** | **Eggs** | **Vegetables** | **Fruits** | **Grains** | **Legumes** | **Nuts and Seeds** |
| **Red Meat** | 0.241 | 0.078 |  |  |  |  |  |  |  |  |  |  |
| **Poultry** | 0.234 | 0.074 | -0.007 |  |  |  |  |  |  |  |  |  |
| **Fish and Seafood** | 0.233 | 0.070 | -0.008 | -0.001 |  |  |  |  |  |  |  |  |
| **Dairy** | 0.152 | 0.150 | -0.090* | -0.082* | -0.081* |  |  |  |  |  |  |  |
| **Eggs** | 0.401 | 0.396 | 0.160* | 0.167* | 0.168* | 0.249* |  |  |  |  |  |  |
| **Vegetables** | 0.032 | 0.052 | -0.209* | -0.202* | -0.201* | -0.119* | -0.369* |  |  |  |  |  |
| **Fruits** | 0.009 | 0.014 | -0.233* | -0.225* | -0.224* | -0.143* | -0.393* | -0.024 |  |  |  |  |
| **Grains** | 0.108 | 0.054 | -0.134* | -0.126* | -0.125* | -0.044* | -0.294* | 0.075* | 0.099* |  |  |  |
| **Legumes** | 0.167 | 0.183 | -0.075* | -0.068* | -0.067* | 0.015 | -0.235* | 0.134* | 0.158* | 0.059* |  |  |
| **Nuts and Seeds** | 0.227 | 0.174 | -0.015 | -0.008 | -0.007 | 0.075* | -0.175* | 0.194* | 0.218* | 0.119* | 0.060* |  |

*p-value <0.05
