# Supplementary material for: Association Between Dietary Protein Intake and Sleep Quality in Middle-Aged and Older Adults in Singapore
Source: Front Nutr. 2022 Mar 9;9:832341. doi: 10.3389/fnut.2022.832341 (PMC8959711; doi:10.3389/fnut.2022.832341)
Supplement: Supplementary file 12 [file Table_12.docx]

**Table S12.** Comparison of Trp:LNAA content between different animal and plant-sourced proteins groups (per 100g) in USDA database

| **Trp:LNAA** | **Mean** | **SD** | **Value Differences between Protein Sources** | | | | | | | | | | |
| --- | --- | --- | --- | --- | --- | --- | --- | --- | --- | --- | --- | --- | --- |
|  |  |  | **Red Meat** | **Poultry** | **Fish Seafood** | **Dairy** | **Eggs** | **Vegetables** | **Fruits** | **Grains** | **Legumes** | **Nuts and Seeds** |  |
| **Red Meat** | 0.041 | 0.008 |  |  |  |  |  |  |  |  |  |  |  |
| **Poultry** | 0.046 | 0.006 | 0.005* |  |  |  |  |  |  |  |  |  |  |
| **Fish and Seafood** | 0.046 | 0.005 | 0.005* | 0.000 |  |  |  |  |  |  |  |  |  |
| **Dairy** | 0.044 | 0.015 | 0.004 | -0.001 | -0.002 |  |  |  |  |  |  |  |  |
| **Eggs** | 0.049 | 0.008 | 0.008 | 0.003 | 0.003 | 0.005 |  |  |  |  |  |  |  |
| **Vegetables** | 0.054 | 0.024 | 0.013* | 0.008* | 0.008* | 0.010* | 0.005 |  |  |  |  |  |  |
| **Fruits** | 0.061 | 0.035 | 0.020* | 0.015* | 0.015* | 0.016* | 0.012* | 0.007* |  |  |  |  |  |
| **Grains** | 0.053 | 0.010 | 0.012* | 0.007* | 0.007* | 0.008* | 0.004 | -0.001 | -0.008* |  |  |  |  |
| **Legumes** | 0.047 | 0.010 | 0.006* | 0.001 | 0.001 | 0.003 | -0.002 | -0.007* | -0.014* | -0.006* |  |  |  |
| **Nuts and Seeds** | 0.053 | 0.013 | 0.013* | 0.008* | 0.007* | 0.009* | 0.004 | -0.001 | -0.007* | 0.001 | 0.006* |  |  |

*p-value <0.05
